# Characterization of PEDOT:PSS Nanofilms Printed via Electrically Assisted Direct Ink Deposition with Ultrasonic Vibrations

**DOI:** 10.3390/molecules28207109

**Published:** 2023-10-16

**Authors:** Yizhen Zhu, Rohan Ravishekar, Tengteng Tang, Banashree Gogoi, Carson Gockley, Sushmitha Venu, Terry L. Alford, Xiangjia Li

**Affiliations:** 1 School for Engineering of Matter, Transport and Energy, Arizona State University, Tempe, AZ 85287, USA; yzhu245@asu.edu (Y.Z.); rravishe@asu.edu (R.R.); ttang32@asu.edu (T.T.); svenu2@asu.edu (S.V.); 2 School of Molecular Sciences, Arizona State University, Tempe, AZ 85287, USA; banashreegogoi135@gmail.com; 3 School for Electrical, Computer and Energy Engineering, Arizona State University, Tempe, AZ 85287, USA; cpgockle@asu.edu

**Keywords:** PEDOT:PSS, conductive nanofilm, perovskite solar cells, electrospray, ultrasonic vibrations

## Abstract

Poly(3,4-ethylenedioxythiophene):poly(styrenesulfonate) (PEDOT:PSS) has emerged as a promising conductive polymer for constructing efficient hole-transport layers (HTLs) in perovskite solar cells (PSCs). However, conventional fabrication methods, such as spin coating, spray coating, and slot-die coating, have resulted in PEDOT:PSS nanofilms with limited performance, characterized by a low density and non-uniform nanostructures. We introduce a novel 3D-printing approach called electrically assisted direct ink deposition with ultrasonic vibrations (EF-DID-UV) to overcome these challenges. This innovative printing method combines programmable acoustic field modulation with electrohydrodynamic spraying, providing a powerful tool for controlling the PEDOT:PSS nanofilm’s morphology precisely. The experimental findings indicate that when PEDOT:PSS nanofilms are crafted using horizontal ultrasonic vibrations, they demonstrate a uniform dispersion of PEDOT:PSS nanoparticles, setting them apart from instances involving vertical ultrasonic vibrations, both prior to and after the printing process. In particular, when horizontal ultrasonic vibrations are applied at a low amplitude (0.15 A) during printing, these nanofilms showcase exceptional wettability performance, with a contact angle of 16.24°, and impressive electrical conductivity of 2092 Ω/square. Given its ability to yield high-performance PEDOT:PSS nanofilms with precisely controlled nanostructures, this approach holds great promise for a wide range of nanotechnological applications, including the production of solar cells, wearable sensors, and actuators.

## 1. Introduction

Solar energy, renowned for its boundless and eco-friendly attributes, is a promising solution to address ever-growing global energy demands. In recent decades, extensive research efforts have been directed toward enhancing the efficiency of solar cells to harness this abundant resource more effectively. Among various types of solar cells, perovskite solar cells (PSCs) have garnered considerable attention from the scientific community due to their cost-effectiveness and impressive power conversion efficiency [1,2]. Generally, a PSC comprises an absorber layer between an electron-transport layer (ETL) and a hole-transport layer (HTL). Each layer plays a pivotal role in the device’s performance, significantly influencing the overall cell efficiency. The ETL requires specific attributes, including a high absorption coefficient, direct bandgap transition, and relatively high carrier mobility of free electrons and holes [3]. Moreover, the HTL facilitates the efficient extraction and transport of positive charge carriers generated when the active layer of the solar cell absorbs light. It aids in maximizing the conversion of light energy into electrical energy, contributing to the overall performance and efficiency of the solar cell [4].

In recent years, poly(3,4-ethylenedioxythiophene):poly(styrenesulfonate) (PEDOT:PSS) has gained significant traction as a promising material for the HTL in PSCs. Importantly, its water processability provides an environmentally friendly and cost-effective edge over other HTL materials. Renowned for its high conductivity, excellent transmittance, and favorable work function, thin HTL PEDOT:PSS nanofilms offer numerous advantages in enhancing the performance of solar cells. The fabrication of thin HTL PEDOT:PSS nanofilms for PSCs traditionally employs various manufacturing methods, including spin coating, spray coating, and slot-die coating [4,5]. Spin coating, a quick and simple technique for producing uniform nano/microlayers, is favored in research and small-scale production environments. Nevertheless, its limitations in batch operations restrict its large-scale applicability [6,7].

In contrast, spray coating is a better-suited method for industrial-scale production. However, challenges such as droplet isolation, surface unevenness, and pinhole formation can adversely affect the resultant nanofilm quality [8]. Another noteworthy manufacturing approach for PEDOT:PSS nanofilms is slot-die coating, a pre-metered deposition technique that holds promise for commercial PSC manufacturing. Minimizing ink waste and enabling precise film thickness control are potentially advantageous for large-scale production [9]. Overall, the electrical conductivity of PEDOT:PSS nanofilms fabricated using current manufacturing approaches can vary substantially, from 10^−4^ to 10^3^ S·cm^−1^, depending on factors such as synthesis conditions, doping additives, and post-processing treatments, posing significant challenges to achieving consistency and reliability in PSC applications [4,5].

Recent developments in the field have demonstrated the potential advantages of incorporating ultrasonic vibrations in the fabrication process of PEDOT:PSS nanofilms. Studies conducted by Gholampour et al. and Kordshuli et al. have shown that the post-treatment of ultrasonic vibrations can enhance the electrical sheet conductivity and surface roughness of spin-coated PEDOT:PSS films, leading to significant improvements in their overall electrical conductivity [10,11]. Despite these advancements, challenges persist in achieving uniformity and density in the fabrication of PEDOT:PSS nanofilms [12,13,14,15]. This study proposes a one-step printing technique called electrically assisted direct ink deposition with ultrasonic vibrations (EF-DID-UV) for producing thin PEDOT:PSS nanofilms for PSCs. This innovative approach integrates ultrasonic vibrations with electrohydrodynamic spraying to ensure even nanoparticle deposition, thereby enhancing the overall characteristics of the nanofilms. We comprehensively examine the impact of ultrasonic vibrations on the properties of thin PEDOT:PSS nanofilms both during and after the electrospray printing process. Key ultrasonic vibration process parameters, including transducer power and frequency, are thoroughly investigated. We aim to provide detailed insights into the quality and compatibility of these nanofilms for PSC applications through a meticulous analysis of the HTL PEDOT:PSS nanofilm nanostructures, interfacial properties, and electrical conductivity. The EF-DID-UV technique offers a scalable and efficient approach to producing high-quality PEDOT:PSS nanofilms, addressing the challenges in uniformity and density that have hindered large-scale manufacturing. Our study drives the advancement of PSC technology and extends its impact to the broader field of wearable electronics, paving the way for a greener and more connected future.

## 2. Results and Discussion

### 2.1. 3D Printing of PEDOT:PSS Nanofilms Using EF-DID-UV

**Figure 1:**
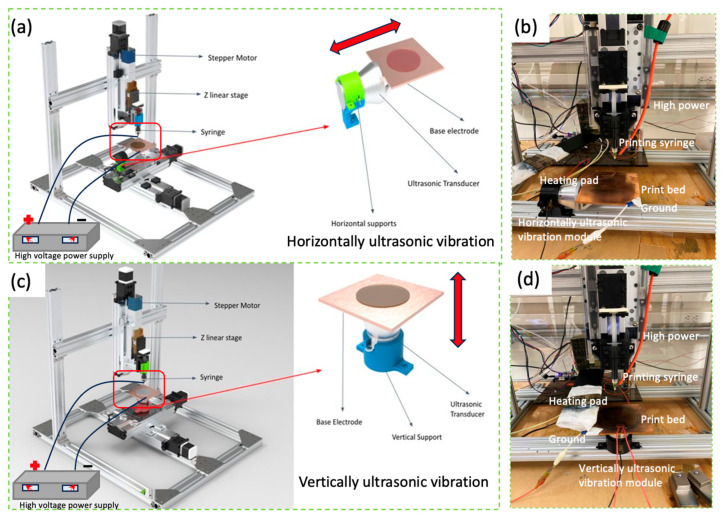
Schematic diagram (**a**) and physical prototype EF-DID-UV machine (**b**) with a horizontal vibration module, and a schematic diagram (**c**) and physical prototype EF-DID-UV machine (**d**) with a vertical vibration module.

A prototype EF-DID-UV machine was designed and built, and the schematic diagram and physical prototype EF-DID-UV machine is shown in Figure 1. In the EF-DID-UV process, we harness electrical fields generated by a high DC voltage to create a fine and controlled aerosol of charged PEDOT:PSS droplets, subsequently deposited onto the ITO substrate to form a nanofilm. The process begins with the formation of a Taylor cone under the metal needle when a high voltage is applied between the needle and the printing bed. By adjusting the applied voltage, we can tailor the shape of the Taylor cone. This cone then emits a PEDOT:PSS liquid jet, generating small and highly charged PEDOT:PSS droplets due to varicose waves on the jet’s surface.

The charges within the conductive printing ink cause the PEDOT:PSS droplets to disperse and form a stable spray under the influence of the applied electrical field. As these charged droplets travel through the electric field toward the ITO substrate, they evaporate, driven by the solvent’s low boiling point. This process results in the formation of highly charged nanoscale PEDOT:PSS particles that are directed toward the ITO substrate under the influence of the electric field. During deposition, the charged PEDOT:PSS nanoparticles undergo rearrangement and self-assembly, driven by Coulombic interactions and van der Waals forces [16]. These forces create well-defined PEDOT:PSS nanoparticles within the nanofilm.

The formation of PEDOT:PSS nanoparticles under an electrical field is influenced by various factors, including the electrical field strength, liquid properties (such as viscosity and surface tension), and ITO substrate characteristics. Our previous work used a metal needle with a nozzle diameter of 90 µm. We applied a voltage difference of 11 kV between the printing needle and the ground copper electrode to create the electrical field [16]. The ITO substrate was positioned at a fixed distance of 40 mm from the needle tip to ensure optimal uniformity. Our previous findings indicated that the optimal PEDOT:PSS deposition duration for desirable film properties for PSCs was 30 s [16]. These specific parameters facilitated the formation of a stable Taylor cone and ensured a consistent deposition stream of PEDOT:PSS nanoparticles during the printing process.

To achieve enhanced uniformity and precision control of the morphology of PEDOT:PSS nanoparticles, we integrated ultrasonic vibrations and electrohydrodynamic deposition for PEDOT:PSS nanofilm fabrication. Both horizontal and vertical ultrasonic vibration modules were tested to evaluate their effects on the nanostructure of printed PEDOT:PSS films. For horizontal vibrations, the top section of the ultrasonic transducer was reinforced by attaching a gusset bracket using epoxy. Initially, a hex screw and nut were used to secure the bracket onto the transducer, but it failed to withstand the increased amplitude of the vibrations, resulting in wear on the surface of the transducer due to intense friction. To address this issue, epoxy was applied as an adhesive to attach the bracket to the transducer firmly. The copper electrode was then affixed to the flat surface of the bracket using epoxy, and a heating pad was secured on top using additional epoxy. Horizontal supports were installed to stabilize the transducer, maintaining the setup in a horizontal orientation. Wires were connected to electrodes of the transducer, linking them to the power supply. A CAD model of the entire system (Figure 1a) was created using SolidWorks, and the prototype machine was constructed for testing (Figure 1b). In terms of vertical vibrations, the copper electrode was securely affixed to the top section of the transducer using epoxy, and the heating pad was then attached on top with epoxy as well. Vertical supports were installed to provide stability, and the entire setup was maintained in a vertical orientation (Figure 1c). Wires were connected from the transducer’s electrodes to the power supply, ensuring proper electrical connections. The physical setup for vertical ultrasonic vibrations is shown in Figure 1d.

Ultrasonic vibrations with various parameters were tested to optimize the printing results for PEDOT:PSS nanofilms using EF-DID-UV. PEDOT:PSS nanofilms were printed under horizontally applied ultrasonic vibrations (H) and vertically applied ultrasonic vibrations (V) to investigate the effect of vibration direction on the deposition characteristics of the PEDOT:PSS nanostructures. Moreover, the vibration amplitude of the transducer was controlled by adjusting the current output from the generator. The maximum allowed current for a single transducer, which corresponds to the full amplitude mode, was set at 0.3 A. Additionally, a half amplitude mode was explored, using a current of 0.15 A, to assess its impact on the fabrication results of PEDOT:PSS nanofilms. In addition, PEDOT:PSS nanofilms were printed with ultrasonic vibrations during (D) and after (P) electrohydrodynamic deposition. In terms of post-ultrasonic vibration application, the PEDOT:PSS ink was deposited on ITO slides normally, and then ultrasonic vibrations were applied for 120 s immediately after deposition. The detailed effects of the ultrasonic vibrations on the printed PEDOT:PSS nanofilms are thoroughly discussed in the following sections.

### 2.2. Influence of Ultrasonic Vibration Direction and Amplitude

**Figure 2:**
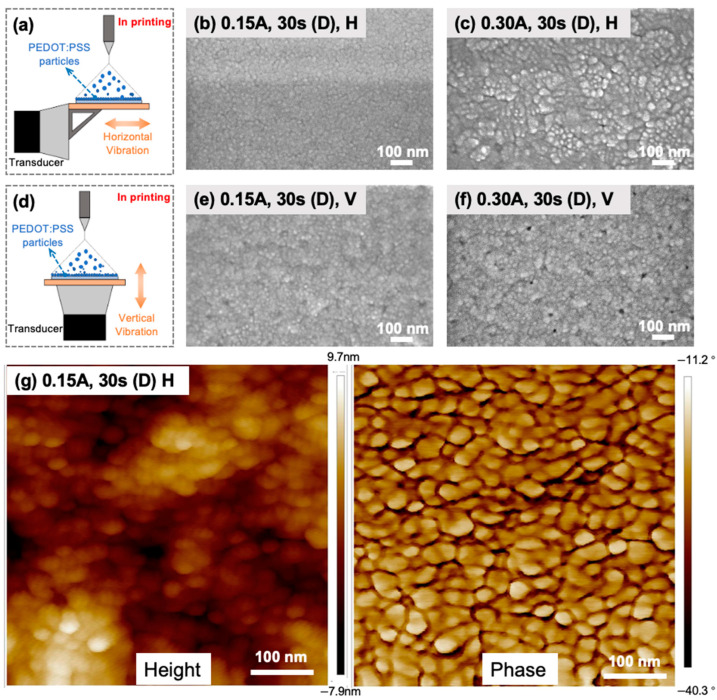
In-printing vibrations. Illustration of (**a**) horizontal vibrations and (**d**) vertical vibrations during the EF-DID-UV printing process. The surface morphology of the nanofilms was fabricated using the following parameters: (**b**) no vibrations; (**c**) 0.15 A current and horizontal vibrations; (**e**) 0.15 A current and vertical vibrations; (**f**) 0.30 A current and horizontal vibrations; (**g**) 0.15 A current and horizontal vibrations. The latter are presented as AFM phase and height images of the PEDOT:PSS nanofilms.

The integration of ultrasonic vibrations and electrohydrodynamic deposition plays a fundamental role in enhancing the uniformity and morphology of the PEDOT:PSS nanoparticle assembly. This process introduces mechanical waves into the system, resulting in several key effects that improve the uniform formation of PEDOT:PSS nanoparticles. To visually explore the influence of ultrasonic vibrations on the morphology of PEDOT:PSS nanofilms, scanning electron microscopy (SEM) images were taken to examine the density, uniformity, and grain size distribution of the printed PEDOT:PSS nanofilms. The impacts of vibration direction and amplitude during electrohydrodynamic deposition on the nanostructure formation of the PEDOT:PSS nanofilms were firstly examined (Figure 2). 

Ultrasonic vibrations play a crucial role in creating the PEDOT:PSS liquid jet emanating from the Taylor cone during electrohydrodynamic spraying. The mechanical agitation induced by the ultrasonic waves effectively breaks down the jet into smaller droplets, leading to a finer and more uniform aerosol (Figure 2a). This horizontal vibration during the deposition mitigates the issue of droplet size variations, resulting in more consistent PEDOT:PSS nanoparticle deposition on the ITO substrate. Remarkably, significant cracks and defects are evident on the PEDOT:PSS nanofilm printed without vibrations, and the surface thickness exhibits non-uniformity (Figure 2b). In contrast, the PEDOT:PSS nanofilm printed under horizontal ultrasonic vibrations (Figure 2a) with a low vibration amplitude (0.15 A) exhibited a uniform distribution of grains that are of identical size and free from cracks and defects (Figure 2c). However, the configuration involving vertical vibrations (Figure 2d) demonstrated a less homogeneous pattern when printing under the same lower amplitude (Figure 2e). This observation is attributed to the fact that horizontal ultrasonic vibrations promote the redistribution and reorganization of PEDOT:PSS nanoparticles on the ITO substrate surface. The mechanical waves encourage the horizontal movement and rearrangement of charged PEDOT:PSS nanoparticles, resulting in more uniform coverage and reduced aggregation (Figure 2a). As a consequence, this effect contributes to the formation of well-defined and evenly distributed PEDOT:PSS nanoparticle structures, ultimately enhancing the overall morphology of the deposited PEDOT:PSS nanofilms (Figure 2c). Additionally, ultrasonic vibrations aid in IPA solvent removal during the deposition process [17]. The mechanical energy from the ultrasonic waves accelerated solvent evaporation, and drying times for the deposited PEDOT:PSS nanoparticles tended to be faster than those for the nanoparticles fabricated without vibration. This rapid drying also prevented excessive spreading and clustering of PEDOT:PSS nanoparticles, contributing to improved density, uniformity, and controlled morphology of the PEDOT:PSS nanofilms. 

Upon increasing the vibration amplitude of horizontal vibrations (current 0.30 A), we observed a significant enlargement in grain size, which was three times larger than the one printed under a low amplitude, consequently leading to the formation of a roughened surface (Figure 2f). Similarly, with vertical vibrations, a further increase in the amplitude led to a doubling of the grain size and the appearance of pore defects on the nanofilm surface (Figure 2g). These observations demonstrated that the movement and rearrangement of charged PEDOT:PSS nanoparticles aligned with the vibration direction. Conversely, vertical vibrations and higher current intensities resulted in a less homogeneous grain distribution, an increased grain size, and the emergence of pore defects, adversely affecting the surface morphology of the nanofilm. In contrast, the utilization of horizontal vibrations with a low amplitude (0.15 A) yielded superior print quality, characterized by a uniform grain distribution and the absence of structural defects. To further analyze the nanofilm’s surface morphology and structures, we conducted an atomic force microscopy (AFM) analysis of the nanofilms printed under horizontal vibrations with a low amplitude. As shown in Figure 2h, the surface profile and topography of the PEDOT:PSS nanofilms printed under these conditions exhibited a smoothening effect on the films with a more refined and controlled morphology and nanostructure.

### 2.3. Influence of Ultrasonic Vibration Modes 

**Figure 3:**
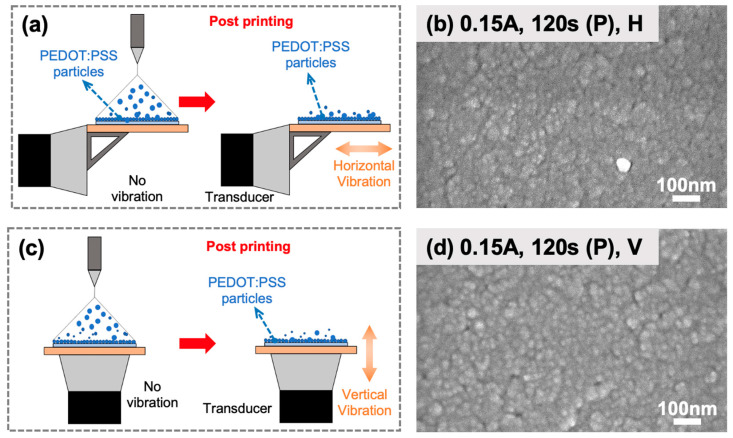
Post-printing treatment with vibrations. Illustration of spreading and clustering of PEDOT:PSS nanoparticles under (**a**) horizontal vibrations and (**c**) vertical vibrations after electrohydrodynamic deposition. Surface morphologies of PEDOT:PSS nanofilms with processing treatment parameters of (**b**) 0.15 A current and post-treatment horizontal vibrations and (**d**) 0.15 A current and post-treatment vertical vibrations.

This post-treatment with ultrasonic vibrations involves subjecting the freshly deposited PEDOT:PSS nanoparticles to additional movement, inducing various effects (Figure 3a,c). The mechanical waves generated during post-treatment vibrations promote interparticle interactions, facilitating the packing of PEDOT:PSS nanoparticles more closely together, leading to an increased film density and a higher packing efficiency. The enhanced packing is beneficial for the nanofilm’s performance in terms of charge transport and conductivity, as closely packed nanoparticles facilitate efficient charge transfer between neighboring particles [18]. Further exploration of the impact of post-treatment vibrations on the morphology of PEDOT:PSS nanofilms revealed several important observations (Figure 3). Nanofilms fabricated with both horizontal and vertical post-treatment vibrations did not exhibit any crack defects, and the nanofilm’s porosity decreased, further improving film density and packing efficiency. A comparison between horizontal and vertical post-treatment vibrations showed that the nanofilms fabricated under horizontal vibrations exhibit an even larger density and a smaller surface roughness (Figure 3b,d).

However, it is essential to note that post-treatment ultrasonic vibrations induced changes in the nanofilm’s surface morphology and roughness. In contrast to vibrations applied during deposition, vibrations applied after deposition resulted in clustering of PEDOT:PSS nanoparticles, leading to an increase in the nanofilm’s surface roughness, as depicted in Figure 2c and Figure 3b. This increase in surface roughness can have implications for the nanofilm performance, as a rough surface hinders charge collection and transport in terms of the HTL for solar cells. In addition, under the same manufacturing parameters, post-treatment vibrations, particularly vertical vibrations, were observed to result in non-uniform surface topography, where the deviation in the grain size is 2.5 times that of the one printed under the same vibrations applied during deposition (Figure 2e and Figure 3d). This suggests that the application of post-treatment vibrations had a disruptive effect on the arrangement and distribution of grains within the PEDOT:PSS nanofilms. The observed increase in the surface roughness and the non-uniformity of the grain distribution can be attributed to mechanical agitation, which induced extra spreading and clustering of PEDOT:PSS nanoparticles. These changes in surface characteristics can influence the PEDOT:PSS film’s electrical conductivity and wettability, which are critical factors affecting the overall performance of the HTL PEDOT:PSS nanofilms in PSCs.

### 2.4. Characterization of PEDOT:PSS Nanofilms

**Figure 4:**
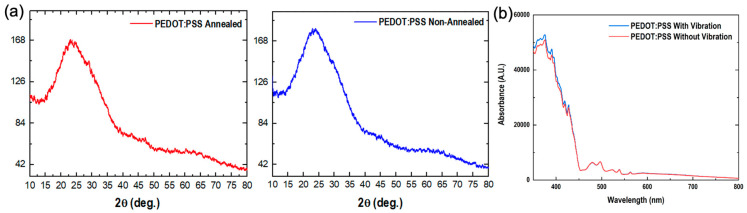
(**a**) XRD patterns of annealed and non-annealed PEDOT:PSS nanofilms and (**b**) the UV absorption spectra of PEDOT:PSS nanofilms printed with and without horizontal vibrations.

Comprehending the fundamental mechanisms of this process is paramount for optimizing the deposition strategy and attaining the desired performance in the PEDOT:PSS nanofilms intended for solar cell applications. To delve into the performance characterization, we conducted X-ray diffraction (XRD) analysis of the printed nanofilms. Figure 4a shows the XRD patterns of both annealed and non-annealed PEDOT:PSS films. Both patterns affirm the amorphous nature of the PEDOT:PSS films. Nevertheless, a subtle distinction in crystallinity is evident between the two samples. The non-annealed PEDOT:PSS film exhibits a more pronounced amorphous character, while the PEDOT:PSS film annealed at 130 °C reveals discernible crystallographic planes, marked by the sharp peaks in its XRD pattern. This improved crystallinity can be attributed to the annealing process, which facilitated coalescence by evaporating the IPA solvent present in the film, with a boiling point of 85 °C. Furthermore, we conducted an analysis of the absorbance spectra for the printed PEDOT:PSS films, comparing those with and without vibration treatment. In the UV absorption spectra of the PEDOT:PSS nanofilms, we observed a reduction in absorption upon applying the vibration treatment (Figure 4b). This decrease is likely a result of improved PEDOT:PSS film quality, as the vibration treatment appears to aid in removing trap holes, leading to enhanced density.

**Figure 5:**
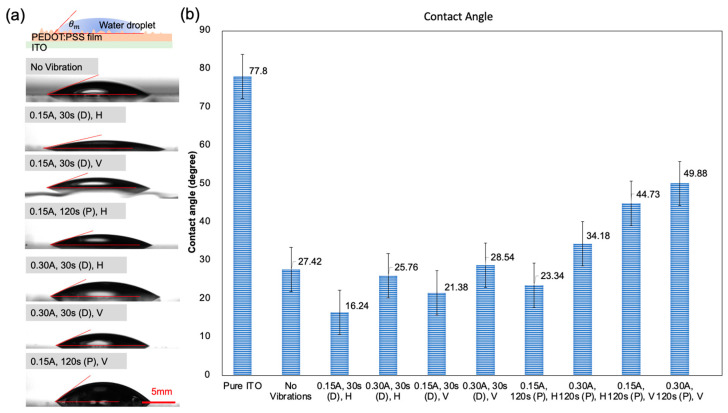
(**a**) Contact angle measurement images and (**b**) contact angle results of deposited PEDOT:PSS nanofilms printed using different vibration process parameters.

The wettability properties of the printed PEDOT:PSS nanofilms were meticulously investigated, as optimizing these properties is crucial for achieving efficient charge extraction and appropriate energy level alignment between the HTL and the active layer, ultimately enhancing the overall performance of the PSC device. The contact angle of water on the printed film was used as an indicator of wettability (Figure 5a). Wenzel’s equation for the apparent contact angle $\theta_{m}$ formed by a liquid wetting a rough surface is given by $\cos\theta_{m}=r\cos\theta_{y}$, where r is the average roughness ratio and $\theta_{y}$ is the intrinsic contact angle [19]. According to the Wenzel model, the apparent contact angle increases when a surface is roughened due to the increased contact area between the liquid and the rough surface [20].

The contact angle testing results aligned well with the SEM analysis in terms of the surface roughness. Specifically, PEDOT:PSS nanofilms deposited under horizontal vibrations with a low amplitude (0.15 A) exhibited the lowest contact angle (16.24°), indicating the smoothest surface roughness (Figure 5b). Contact angles of films fabricated under vertical vibrations were consistently larger than those fabricated under horizontal vibration, indicating the larger surface roughness. Moreover, nanofilms fabricated with vibrations during deposition showed smaller contact angles compared to those fabricated with post-treatment vibrations of the same amplitude. Notably, the contact angles of PEDOT:PSS nanofilms fabricated with vibrations during deposition were all smaller than 30°, with only the contact angles of the nanofilms fabricated under vertical vibrations with a large amplitude exceeding the one without vibrations. In contrast, the post-treatment vibration modes displayed a wide range of contact angle values from 23.34° to 49.88°, with only the contact angles of PEDOT:PSS nanofilms fabricated with the low amplitude horizontal post-treatment vibrations being smaller than those of nanofilms fabricated without vibrations (Figure 5b). An interesting observation was that post-treatment vibrations with a higher amplitude did not improve the printing quality. Larger amplitudes of vibration induced higher mechanical agitation, leading to additional spreading and clustering of PEDOT:PSS nanoparticles, resulting in a roughened surface.

In summary, the results indicate that the amplitude and direction of vibrations play a significant role in the wettability properties of the printed PEDOT:PSS nanofilms. Low-amplitude vibrations during deposition exhibit better wettability compared to films deposited without vibrations, with the amplitude impacting the deposition process and affecting the surface characteristics of the nanofilms. 

**Figure 6:**
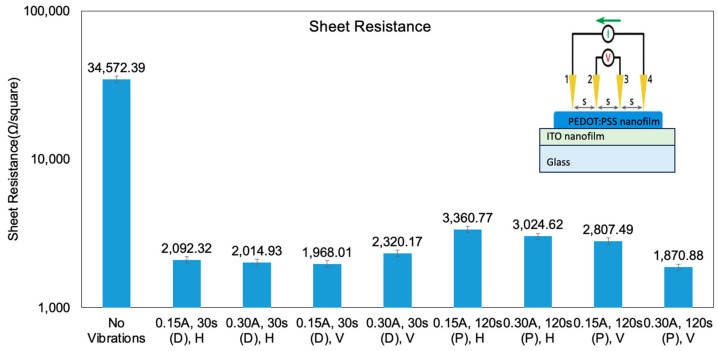
Sheet resistance results of PEDOT:PSS nanofilms fabricated under ultrasonic vibrations with different parameters.

The 4-point probe (4PP) test is a commonly employed method for measuring sheet resistance. In the 4PP test, a current is applied between probe 1 and probe 4, while the voltage is measured between probe 2 and probe 3 within the same probe configuration as depicted in Figure 6. By applying Ohm’s law, the sheet resistance can be calculated based on the voltage generated by the current passing through the film. Analysis of the sheet-resistance data reveals significant improvements when ultrasonic vibrations are employed during both the deposition and post-treatment stages. Notably, the PEDOT:PSS nanofilms deposited without vibrations exhibit the highest sheet resistance, resulting from low density with internal holes and a non-uniform grain size. Generally, ultrasonic vibrations during deposition significantly improve the electrical conductivity compared to post-treatment ultrasonic vibrations. However, among all the tests, full-amplitude post-treatment vertical ultrasonic vibrations, which represent the most intensive vibration mode, exhibit the smallest sheet resistance (1871 Ω/square) (Figure 6). This could be attributed to the largest film thickness since vertical vibrations with full amplitude after deposition cause particle bouncing, influencing the film thickness.

Similarly, the sheet resistances of the PEDOT:PSS nanofilms fabricated with ultrasonic vibrations during deposition are smaller than those fabricated with post-treatment ultrasonic vibrations, which aligns with the trend observed in the wettability analysis (Figure 5). Ultrasonic vibrations during deposition promote fast packing of PEDOT:PSS nanoparticles, and the mechanical energy from the ultrasonic waves accelerates IPA evaporation and the complete removal of residual IPA solution. This process enhances the PEDOT:PSS film’s purity and electrical conductivity. PEDOT:PSS nanofilms fabricated under horizontal vibrations generally exhibit a larger sheet resistance than those fabricated under vertical vibrations, both during and after the printing process (Figure 6). This difference in sheet resistance could be attributed to the fact that PEDOT:PSS nanofilms fabricated under horizontal vibrations have a much thinner nanofilm thickness compared to those fabricated under vertical vibrations.

Overall, the sheet resistance data confirm the considerable impact of ultrasonic vibrations on improving the electrical conductivity of the deposited PEDOT:PSS nanofilms. These findings highlight the importance of carefully considering ultrasonic vibration parameters and orientations in the deposition process to optimize PEDOT:PSS film quality. Further characterization of the nanofilms, including composition analysis and surface profiling, will be conducted to gain deeper insights into the effects of vibrations during deposition for further improvement of the HTL in PSCs.

## 3. Material and Method

### 3.1. Printing Ink Preparation 

The printing ink was formulated by blending water-based PEDOT:PSS solution (Clevios™ P VP AI 4083 from Ossila) with semiconductor-grade isopropyl alcohol (IPA) in a vial. The volume ratio of the mixture was set at 3:7 (*vol*/*vol*). Pipettes with a volume of 1000 µL were used for this task. To eliminate larger undispersed PEDOT:PSS particles, the solution was passed through a 0.45 µm filter. The resulting polymer ink was then loaded into a syringe, ready for printing onto ITO substrates. The ITO substrates (purchased from MXBAOHENG via Amazon) were precisely cut into rectangular slides of 25 mm × 75 mm. These ITO slides exhibit sheet resistances ranging from 10 to 15 Ω/square, and the water contact angle on the surface of ITO slides measures 77.8°. The slides were ultrasonically cleaned with IPA to ensure surface cleanliness. Subsequently, the substrates underwent a 15-min ozone cleaning process in an Ossila UV Ozone Cleaner to eliminate micro/nano-impurities. 

### 3.2. EF-DID-UV

For electrically assisted direct ink deposition (EF-DID), the printing process involved loading the printing inks directly into a 5 mL plastic syringe (purchased from BD Medical, Franklin Lakes, NJ, USA). The syringe’s plunger was connected to a mechanical linear actuator with a 40 mm movement range and 1 μm resolution (purchased from Parker-Hannifin Corporation, Cleveland, OH, USA) for material extrusion. A stainless steel needle with a blunt end (Bstean 32G 1⁄2 inch, 0.09 mm inner diameter, and 0.25 mm outer diameter) was utilized for material deposition [16]. To generate the required electrical field during electrohydrodynamic deposition, a Hipotronics HD 125A (purchased from Accusource, Gainesville, GA, USA) was employed as the high-direct-current (DC) voltage power supply with a range of 0 to 35 kV. A 99.9% pure copper sheet was used as both the substrate and the ground electrode for electrohydrodynamic-assisted direct ink deposition. When a high voltage was applied, the liquid meniscus at the tip of the deposition needle formed a semi-spherical shape, resulting in the induction of an electrical field around the nozzle [16,21]. To initiate the printing process and ensure proper solution flow, the plunger was initially moved down by 0.4 mm to eliminate any air gap near the needle base. The print speed was adjusted using the G codes, and the deposition time was controlled via the Arduino code.

A Derui digital ultrasonic generator with an output of 600 W and a frequency of 28 kHz was employed to generate ultrasonic vibrations. A relay was connected to the ultrasonic transducer and the Arduino control board. Before integrating the vibrational setup into the EF-DID printing setup and prior to installing the heating pad, a thorough test of the vibration module was conducted. This involved adding a few drops of water to the surface of the copper electrode and then adjusting the current supply to vary the amplitude of vibrations. The behavior of the water droplet was carefully observed as the amplitude of vibrations increased. Eventually, beyond a certain threshold, the droplet evaporated due to the heat generated from friction. This successful test confirmed the effective functioning of the ultrasonic transducers. To control the shutter and vibration module, an Arduino Uno control board with a 2-channel DC 5 V relay module was utilized. The microcontroller was appropriately programmed to ensure the vibrations were implemented accurately during and after deposition. Additionally, a Duet2 control board was used to control the ink deposition and annealing processes after printing. Either horizontal or vertical vibrations were applied during or after the electrohydrodynamic deposition process of the PEDOT:PSS nanoparticles. To prevent any displacement of the ITO slide during the application of ultrasonic vibrations, stainless steel clamps were employed. These clamps securely held the ITO slide in place, maintaining its alignment along the central axis of the deposition.

### 3.3. Post-Heat Treatment

After the completion of the printing process, the clamps were released, and the printed ITO slide was carefully removed and placed on a ceramic hot plate for post-processing annealing. Each slide was subjected to a temperature of 100 °C for a duration of 15 min. Subsequently, printed slides were stored in Petri dishes for further testing and analysis. To ensure the uniformity of PEDOT:PSS films and prevent the formation of pinholes, the printing substrate was not heated during the printing process. This allowed for better control and consistency during the subsequent post-heat treatment. The ALT (American Laboratory Trading) VWR 18 × 18 cm ceramic hot plate stirrer was utilized to anneal the PEDOT:PSS nanofilms printed on the substrate. 

### 3.4. Morphology, X-ray Diffraction Analysis, Absorbance Spectra, and Electrical Conductivity Characterization

The Zeiss Auriga SEM was utilized to capture high-resolution images of the surface morphology of the EF-DID-UV-printed PEDOT:PSS nanofilms. The root-mean-square (RMS) roughness of the PEDOT:PSS films was characterized using an AFM (SPM Bruker Multimode (MM8)). The crystallinity, phase composition, and structural properties of both annealed and non-annealed PEDOT:PSS nanofilms were assessed through XRD analysis using a Bruker AXS-D8 instrument. The XRD analysis involved a scanning step of 0.0054332°, with a 4.845-s duration per step, and a 2θ range spanning from 10° to 90°. During this process, X-rays were directed at the nanofilms and diffracted at various angles, with a detector recording the diffracted X-rays as a function of the diffraction angle (2θ), resulting in peaks corresponding to distinct crystal planes within the sample. Furthermore, absorbance spectra of the PEDOT:PSS nanofilms, both with and without horizontal vibration treatment, were obtained using a Perkin Lambda 950 spectrometer. To ensure that the incident light remained within the sample area, an adjustable mask was employed, and an Autozero procedure was executed with the mask in position. For the absorbance measurements, all samples were securely affixed to the mask and tested over a wavelength range from 350 nm to 800 nm, with a wavelength interval set at 5 nm. Additionally, the KLA Tencor Omnimapper 4-Point Probe was employed to measure the electric conductivity of the nanofilms.

### 3.5. Wettability Evaluation

The surface contact angle of a water droplet on the printed PEDOT:PSS nanofilms was measured using a SINDIN contact angle meter. Water was chosen for the measurement, as the PEDOT film is intended for use as the HTL in a PSC, which primarily operates in ambient environments. PEDOT:PSS is a hydrophilic polymer, and a smaller contact angle indicates better deposition of PEDOT:PSS onto the ITO substrate, resulting in a high-quality film. Lower contact angles also contribute to reduced diffraction and reflection from the PEDOT film, indicating increased efficiency of the overall solar cell device. To perform the measurement, ITO slides with printed PEDOT:PSS nanofilms were mounted on a glass cube positioned on the instrument’s base. The light amplitude from an LED was adjusted to ensure clear visibility of the ITO substrate and the glass block. A syringe containing water was used to place a microdroplet on the PEDOT:PSS nanofilm surface, while a camera mounted on the device captured snapshots of the droplet. The camera was activated through software installed on a personal laptop, with a USB connection to power it on. Another USB pen drive was utilized to decrypt the software required to operate the camera and conduct the contact angle test. Once the snapshots were captured, one was selected, and an automatic fitting operation was performed. This operation calculated and displayed the resulting contact angle (in degrees) on the screen, providing a quantitative measurement of the surface contact angle of the water droplet on the PEDOT:PSS nanofilm. 

## 4. Conclusions 

The innovative 3D-printing technique, electrically assisted direct ink deposition with ultrasonic vibrations (EF-DID-UV), proved to be successful in fabricating PEDOT:PSS nanofilms for the ETL of PSCs. This integration allowed for precise uniform deposition of PEDOT:PSS nanoparticles onto the ITO substrate, leading to improved film characteristics. Comprehensive examination was conducted to assess the effects of ultrasonic vibrations during and after deposition, with specific attention given to key parameters such as transducer power and frequency. Microstructural analysis of the fabricated PEDOT:PSS films after a post-annealing process revealed that horizontal vibrations with a low amplitude (0.15 A) yielded the most uniform distribution of PEDOT:PSS nanoparticles, exhibiting a film free from cracks and obvious pores. To further understand the impact of ultrasonic vibrations on film properties, evaluations were conducted on properties such as wettability and electrical conductivity. These assessments provide valuable insights into the characteristics and performance of the films. The evaluation of film nanostructures, interfacial properties, and electrical conductivity contributes to a comprehensive understanding of the films’ quality and suitability for PSC applications. Notably, PEDOT:PSS nanofilms printed under horizontal ultrasonic vibrations with a low amplitude (0.15 A) showed the best performance, with a contact angle of 16.24° and a sheet resistance of 2092 Ω/square. Moving forward, our research will delve deeper into the influence of ultrasonic vibrations on printing quality by varying the vibration on/off-interval frequency. Additionally, we plan to apply the EF-DID-UV process to print the perovskite layer of PSCs, allowing us to study the performance of the resulting solar cells and further optimize their efficiency. These endeavors are part of our ongoing efforts to advance the mass production of PSCs and unlock the full potential of solar energy as a sustainable power source.

## Data Availability

Not applicable.
